# The push-out bond strength of calcium silicate-based endodontic sealers

**DOI:** 10.1186/s13005-018-0170-8

**Published:** 2018-08-20

**Authors:** David Donnermeyer, Pia Dornseifer, Edgar Schäfer, Till Dammaschke

**Affiliations:** 1 Department of Periodontology and Operative Dentistry, Westphalian Wilhelms-University, Albert-Schweitzer-Campus 1, building W 30, 48149 Münster, Germany; 2 Central Interdisciplinary Ambulance in the School of Dentistry, Albert-Schweitzer-Campus 1, building W 30, 48149 Münster, Germany

**Keywords:** BioRoot RCS, Calcium silicate-based sealer, Endo CPM sealer, Epoxy resin sealer, Push-out bond strength, Total fill BC sealer

## Abstract

**Background:**

The aim was to compare the dislodgement resistance of calcium silicate-based sealers (Total Fill BC Sealer, Endo CPM Sealer, BioRoot RCS) with an epoxy resin-based sealer (AH Plus).

**Methods:**

The root canals of 80 single-rooted human teeth were instrumented with F360 up to size 45.04. All canals were obturated using matching gutta-percha cones according to the single-cone technique in combination with one of the mentioned sealers (*n* = 20 per group). After eight weeks of incubation (37 °C, 100% humidity), the roots were embedded in resin. Starting with a distance of 7 mm from the apex, four slices of 1 mm thickness were cut. Dislodgement resistance was measured using a universal testing machine and the push-out bond strength was calculated. Specimens were examined under 4×-magnification to determine the mode of bond failure. Statistical analysis was performed using ANOVA and Student-Newman-Keuls-test.

**Results:**

Regarding the pooled data of all sections, the push-out bond strength of AH Plus was significantly higher than the push-out bond strength of all calcium silicate-containing sealers (*P* < 0.05). Out of all calcium silicate-based sealers, Total Fill BC Sealer showed the highest push-out bond strength (*P* < 0.05). BioRoot RCS had significant higher push-out bond strength than Endo CPM Sealer (*P* < 0.05). Nearly the same results were found for all four sections. BioRoot RCS only differed significantly from Endo CPM Sealer in the third section (*P* < 0.05).

**Conclusions:**

The push-out bond strength of the investigated calcium silicate-based sealers was lower than of AH Plus. Total Fill BC showed the highest push-out bond strength of the calcium silicate-based sealers.

## Background

The connection between the root canal wall and the root canal filling core material is established by the endodontic sealer. A strong and long-lasting link between the root canal wall and the filling is one aspect of the prevention of root canal infection caused either by regrowth of microorganisms or newly gained infection due to coronal or apical leakage [1, 2]. The bacteria-tight seal of the root canal established by the endodontic sealer is, therefore, a major aspect when evaluating the properties of different sealers [3]. Dislodgement resistance – also called push-out bond strength (POBS) – is regarded as a relevant prognostic factor to evaluate the link of a root canal sealer to the canal wall and the core material [4].

Endodontic sealers based on tricalcium silicate or containing calcium silicate formulations were recently introduced with a view to transferring the well-documented biocompatibility and bioactivity of di- and tricalcium silicate cements to root canal sealers. The release of calcium hydroxide from di- and tricalcium silicate cements due to hydration and the contact with phosphate from tissue fluids leads to a precipitation of calcium phosphate or calcium carbonate on the material’s surface [5, 6]. Also, the formation of hydroxyapatite on a calcium silicate sealer’s surface after contact with phosphate has been reported [6]. This is the reason for the bioactive potential of tricalcium and dicalcium silicate materials and sealers [7]. Furthermore, calcium silicates form an interfacial layer at the dentin wall denoted as “mineral infiltration zone”. The alkaline caustic effects of the calcium silicate cement’s hydration products degrade the collagenous component of the interfacial dentin [8]. This degradation leads to the formation of a porous structure that facilitates the permeation of high concentrations of Ca^2+^, OH^−^, and CO_3_^2−^ ions, leading to increased mineralization in this region [8, 9]. This chemical interaction at the interfacial dentin along with a micromechanical interaction by tag-like structures is mainly the reason for measurable adhesion between calcium silicate-based materials and dentin [8, 10].

Lately, different calcium silicate-based sealers were introduced. Total Fill BC Sealer (FKG, La Chaux-de-Fonds, Switzerland) is a monophasic sealer and contains calcium phosphate monobasic and tricalcium silicate. To allow setting, external fluid supply (e.g. tissue fluid) is required [11]. The formation of hydroxyapatite on its surface [12] has been reported. This sealer is also distributed under the brand names EndoSequence BC Sealer (BUSA, Savannah, USA) and iRoot SP (Innovative BioCeramix, Vancouver, Canada). Endo CPM Sealer (Egeo, Buenos Aires, Argentina) is another calcium silicate-based sealer. The powder mainly contains tricalcium silicate, tricalcium oxide, and tricalcium aluminate. The liquid consists of saline solution and calcium chloride [13]. Good sealing properties [13], as well as the ability to release calcium ions, have been reported [14]. BioRoot RCS (Septodont, St. Maur-des-Fossés, France) is the newest development of a bioceramic sealer. The powder mainly consists of tricalcium silicate, povidone, and zirconium dioxide. The liquid is an aqueous solution of calcium chloride with polycarboxylate [6]. Good biocompatibility was reported for Total Fill BC Sealer [15] and BioRoot RCS [15–18].

AH Plus (Dentsply, Konstanz, Germany) is a conventional epoxy resin-based root canal sealer and is widely used and well investigated [2, 19]. Therefore, it was used as the control group in this study.

Though there is some data about the interfacial interaction between calcium silicate-based sealers and dentin, relevant data about the dislodgement resistance of calcium silicate-based sealers are currently only available for Total Fill BC Sealer. Only one study was found for Endo CPM Sealer concerning dislodgement resistance [20] and no data was available for BioRoot RCS. Therefore, the aim of this study was to evaluate the dislodgement resistance of Total Fill BC Sealer, Endo CPM Sealer, and BioRoot RCS under a clinical setup. The null hypotheses were:

(i) there are no differences between these calcium silicate-based sealers and the epoxy resin based sealer AH Plus regarding push-out bond strength
(ii) there are no differences between these calcium silicate-based sealers and the epoxy resin based sealer AH Plus regarding the mode of failure.

## Methods

Eighty human mandibular premolars with only one straight root canal (curvature < 5°) were included. All roots were observed with a stereomicroscope under 20×-magnification (Expert DN, Müller Optronic, Erfurt, Germany) to exclude cracks, caries or previous root canal treatment. Only single-rooted teeth with a single canal and a single apical foramen were included. This was verified by viewing their buccal and proximal radiographs. The radiographs were also used to check the curvature of the root canals using an imaging software (ImageJ, Wayne Rasband, NIH, MD, USA). The working length was obtained by measuring the length of the initial instrument (K-Files ISO 10 (VDW, Munich, Germany)) at the major apical foramen minus 1 mm. All teeth were cut in a way that a working length of 18 mm was established. Patency of the canal was determined with K-files ISO 10 (VDW). Only teeth whose canal width near the terminus was approximately compatible with ISO 15 were included. This was examined with silver points ISO 15 and 20 (VDW). All root canals were instrumented with F360 files (Brasseler, Lemgo, Germany) using the torque-limited electric motor VDW.Silver (VDW) with the settings according to the manufacturer’s instructions. The F360 file sequence was used up to size 45 with a constant taper of 4% in every root canal. The instruments were used in slow pecking motion with an amplitude of less than 3 mm. The flutes of the instrument were cleaned after three in-and-out movements (pecks) and the root canal was irrigated with 2.5 ml NaOCl 3%. After instrumentation, the canals were irrigated with 2.5 ml NaOCl 3% and 5 ml EDTA 17% as the final irrigant to remove the smear layer using a 30 g open-ended needle (NaviTip, Ultradent, South Jordan, UT, USA). The needle was inserted as deep as possible into the canal without binding. Irrigation was performed with a volume of 1 ml per minute. Finally, the canals were dried with paper points.

The specimens were randomly divided into four groups (*n* = 20). Root canals of all groups were obturated using matching F360 gutta-percha cones size 45.04 (Brasseler). The following groups were established: Group A: Total Fill BC Sealer; Group B: Endo CPM Sealer; Group D: BioRoot RCS; control Group D: AH Plus. All sealers were mixed according to the manufacturers’ instructions and were applied with a 20G intracanal tip (Transcodent, Kiel, Germany) from a syringe into the canal to ensure homogeneous obturation. Root canal obturation was performed according to the single-cone technique using gutta-percha cones matching the F360 system. Before inserting the gutta-percha cone into the root canal, it was covered with sealer. Following obturation, a heated plugger was used to remove the coronal excess gutta-percha with no further vertical compaction. The teeth were radiographed in buccal and proximal view to verify correct obturation. The canal orifices were restored with Cavit G (3 M ESPE, Seefeld, Germany). The teeth were stored in an incubator at 37 °C and 100% humidity for 2 months as it is known that the setting reaction of calcium silicate cement might continue for 21 d [21] to more than a month [22]. All treatment procedures were carried out by the same operator who was proficient in the obturation technique used.

The roots were embedded into acrylic resin vertically (Technovit 4071, Heraeus Kulzer, Hanau, Germany) and sectioned horizontally with a 0.25 mm-low-speed saw (Leitz, Wetzlar, Germany) under permanent water-cooling between a distance of 7.00 mm and 11.75 mm from the apex (Table 1). Thus, four slices of 1 mm thickness were obtained representing the middle third of the root. This resulted in a total of 80 specimens per group. The sample size was calculated with a power calculation for one-way ANOVA (power = 0.8; effect size = 0.25; significance level = 0.05; number of groups = 4) resulting in recommended sample size of minimum 44.6 samples per group. In the case that sectioning of the teeth revealed an oval canal, a canal with isthmuses, or voids in the obturation the whole set of four specimens per root was discarded and replaced by a new set which was gained from an additionally enclosed tooth. This occurred in only 3 teeth.

**Table 1:** Push-out Bond Strength of all sealers tested in N/mm^2^

	Overall		Section “Background” (10.75–11.75 mm from apex)		Section “Methods” (9.5–10.5 mm from apex)		Section “Results” (8.25–9.25 mm from apex)		Section “Discussion” (7.0–8.0 mm from apex)	
	Mean	SD	Mean	SD	Mean	SD	Mean	SD	Mean	SD
Total Fill BC Sealer	3.52^b^	1.41	2.95^b^	1.06	3.84^b^	1.49	3.42^b^	0.92	3.89^b^	1.88
Endo CPM Sealer	1.60^d^	0.83	1.58^c^	0.75	1.53^c^	0.94	1.47^d^	0.79	1.82^c^	0.85
BioRoot RCS	2.31^c^	1.31	1.96^c^	1.20	2.08^c^	1.25	2.76^c^	1.30	2.44^c^	1.43
AH Plus	7.03^a^	2.41	6.12^a^	1.48	6.37^a^	1.74	7.01^a^	1.80	8.62^a^	3.42

*SD* standard deviation. Values with different superscript letters in each column were statistically different at *p* = 0.05 (ANOVA and Student-Newman-Keuls test)

For the dislodgement resistance measurement and mode of failure evaluation, the specimens were coded by the supervisor, who was the only person being aware of the allocation of the coded specimens to the groups. The specimens were placed in a metallic jig with a hole underneath to allow the canal filling material to fall from the canal after dislodgement. A standard size plunger with a tip of 0.6 mm diameter was used to apply the vertical load onto the gutta-percha core of the filling. The diameter of the plunger tip was dimensioned according to the gutta-percha point diameter at 6 mm from the tip to ensure an equal distribution of the load on 60% to 85% of the gutta-percha cone diameter without touching the sealer phase of the root canal filling. The vertical load was applied in an apical to coronal direction and generated by a universal testing machine (Lloyd LF Plus, Ametek, Berwyn, Pennsylvania, USA) at a speed of 1 mm per minute. A graph of the applied load was generated by a software (Nexygen, Ametek, Berwyn, Pennsylvania, USA) and failure of the bond was automatically determined when the graph showed an abrupt reduction of load. This failure load was recorded in Newton (N).

The lateral surface of the root canal of each specimen was calculated by the truncated cone formula *M* = (*R* + *r*) ⋅ *π* ⋅ *m*. The push-out bond strength of each specimen was then calculated and expressed in N/mm^2^ (equivalent to MPa).

**Fig. 1:**
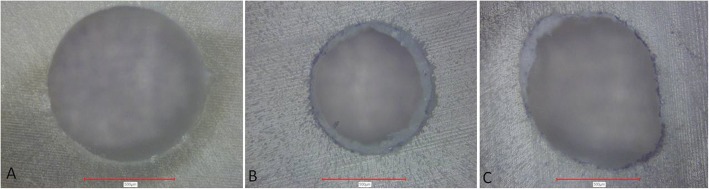
Images obtained by laser microscopy at 4×-magnification for analysis of the mode of failure; examples for adhesive (**a**), cohesive (**b**) and mixed (**c**) failure types are given

After dislodgement of the root canal filling photographs of each specimen were taken with a laser microscope (VK-X100, Keyence, Osaka, Japan) under 4×-magnification. The photographs were separately evaluated by two blinded operators and the mode of failure was recorded. If disagreement existed a joint meeting of all authors was made until a consensus was reached. There were three possible categories: adhesive failure (no material left on canal wall), cohesive failure (material present on entire canal wall) and mixed failure (material in patches on canal wall) (Fig. 1).

Statistical analysis of POBS values was performed using ANOVA and Student-Newman-Keuls-post-hoc-test (*p* < 0.05) as data were distributed normally according to the Kolmogorov-Smirnov test (*p* < 0.05).

## Results

All specimens had measurable adhesion to the root dentin and no premature failure occurred. Pooling the data of the four sections, AH Plus revealed significantly higher dislodgement resistance than all calcium silicate-based sealers (*p* < 0.05). Among the calcium silicate-containing sealers Total Fill BC Sealer showed the highest push-out bond strength values (*p* < 0.05). BioRoot RCS produced significantly higher POBS values than Endo CPM Sealer (*p* < 0.05) (Table 1).

With regards to the different sections (Table 1), Total Fill BC Sealer showed significantly higher POBS values in the first, in the second and in the fourth section than BioRoot RCS and Endo CPM Sealer (*p* < 0.05) while no significant differences were found between BioRoot RCS and Endo CPM Sealer (*p* > 0.05). The third section revealed the same results as the overall results.

**Fig. 2:**
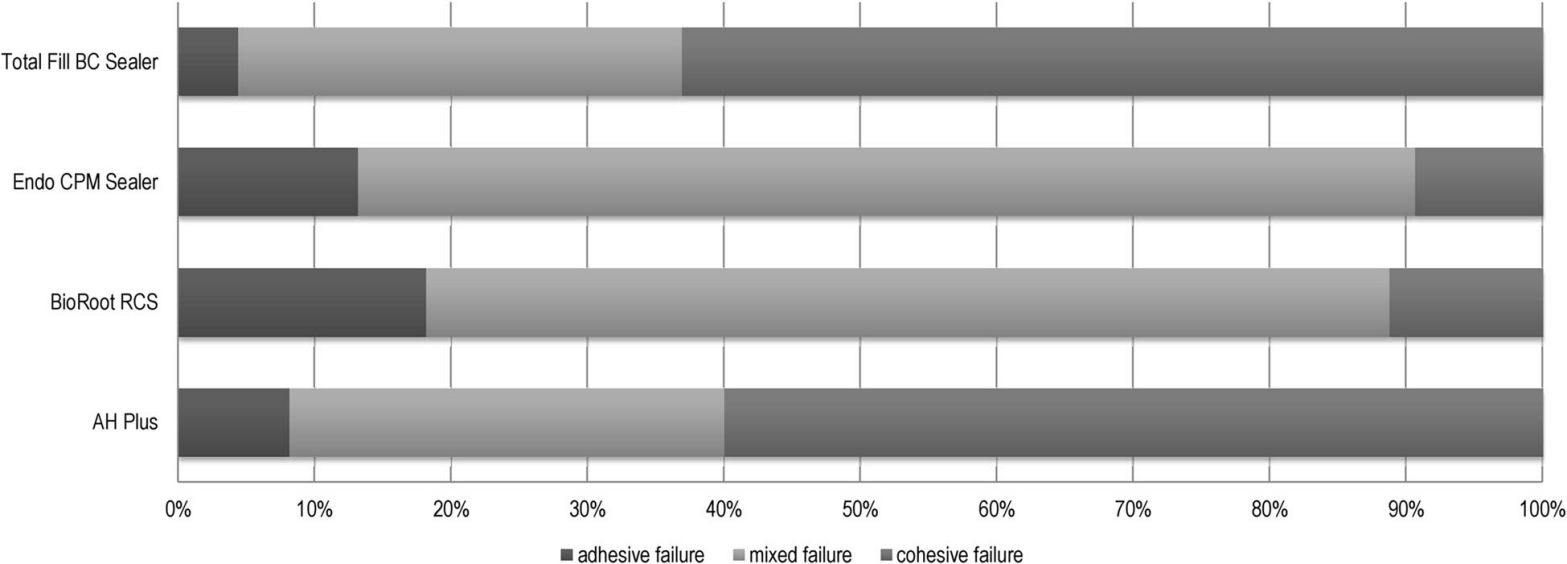
Distribution of failure modes (in %)

The results of the mode of failure analysis are shown in Table 2 and Fig. 2. AH Plus and Total Fill BC Sealer predominantly showed cohesive failure modes whereas BioRoot RCS and Endo CPM Sealer mainly displayed mixed failure modes. For BioRoot RCS and Endo CPM Sealer adhesive failure was the second most common failure mode.

**Table 2:** Mode of Failure during dislodgement of all sealers tested (in %)

Sealer	Mode of failure		
	Adhesive	Mixed	Cohesive
Total Fill BC Sealer	4.4	32.5	63.1
Endo CPM Sealer	13.1	77.5	9.4
BioRoot RCS	18.1	70.6	11.2
AH Plus	8.1	31.9	60.0

## Discussion

A selection of calcium silicate based sealers was investigated for their POBS and failure mode during dislodgement. The push-out test has been widely used to evaluate the dislodgement resistance of root canal filling materials [23]. Statistically significant differences were found between AH Plus and the calcium silicate based sealers. None of the calcium silicate based sealers reached the dislodgement resistance of AH Plus (Table 1). Furthermore, statistically significant differences were found between the calcium silicate based sealers. Differences in the mode of failure were found between AH Plus and some of the calcium silicate-based sealers as well as between the calcium silicate-based sealers. Thus, the null hypotheses (i) and (ii) were rejected.

The investigation of POBS is a commonly used approach to evaluate the dislodgement resistance of a sealer. Within the limitations of the POBS investigation, the bond of the sealer either to the root canal wall or to the core material is assessed with the interaction of a sealer with root dentin being an extraordinarily important factor for the success of endodontic procedures [24]. Although widely used, many different testing protocols have been established [25]. The most crucial step of the experimental setup is the ratio of the pin diameter and the specimen’s diameter [4, 24]. A ratio of less than 0.6 was reported to influence the POBS in both studies. *Chen* et al. [24] also mentioned that a ratio higher than 0.85 may influence the POBS test, which could not be approved by *Pane* et al. [3]. In the present study, the pin diameter was designed to be within this range. Moreover, different experimental protocols that were established in the past concerning root canal preparation (diameter and taper), root canal obturation (cold versus warm obturation techniques), the tooth type and portion, slice thickness, load velocity, and other parameters can be an explanation for the variability in results [23, 26]. In the present study, a reproducible instrumentation was sought by a preparation up to size 45 with a continuous taper of .04. Regarding obturation techniques, lateral compaction and warm vertical compaction may exert a certain impact on the dislodgement resistance [27, 28] and are less reproducible than the single-cone technique. Therefore, the single-cone obturation using matching gutta-percha cones was performed in the present study. Recently, a standardization of the push-out test was demanded to investigate special questions regarding the sealer-dentin-interface. In these studies, the root canal filling was established without the use of a core material such as gutta-percha [28, 29]. Despite a possible confounding factor due to the use of a core material, the present study was designed to provide information about the dislodgement resistance of calcium silicate-based sealers in a clinically orientated setup. Therefore, the sealers were used in a manner that is recommended by the manufacturer and commonly used by clinicians.

In accordance with the present data superior POBS has been reported for AH Plus compared to calcium silicate-based sealers [20, 26, 30–34]. POBS values differ depending on the different protocols (preparation taper, obturation technique, plunger dimensions) used in these studies and are therefore not comparable. Total Fill BC Sealer has been reported to possess inferior POBS compared to AH Plus [26, 30–32], which corroborates the results of the present study. However, another study reported no differences regarding the POBS of AH Plus and Total Fill BC Sealer [33]. In summary, the present results are in major compliance with the current data. In contrast to the present study, Endo CPM Sealer achieved higher POBS values than AH Plus in another investigation [20]. In that study, root canal preparation was performed with a taper of only 2% and obturation was performed by lateral compaction [20]. These differences in the experimental setup might be an explanation for the contradictory findings. No further data regarding the dislodgement resistance of Endo CPM Sealer are currently available. For BioRoot RCS no data regarding POBS have been published yet. In the present study, BioRoot RCS showed inferior POBS than AH Plus and Total Fill BC Sealer, but superior values compared to Endo CPM Sealer.

On the basis of the data available it can be concluded that AH Plus has high resistance to dislodgement in general. The covalent bonds between the epoxy resin and the amino groups of the dentinal collagen [35, 36] may result in a stronger link of AH Plus to dentin compared to the interaction of calcium silicates to dentin. The micromechanical interaction between the root canal wall and the calcium silicate based sealer by the tag-like structures and the chemical interaction by the “mineral infiltration zone” [8] establish a weaker link to the dentin compared to epoxy resins. This observation also corroborates the clinically orientated findings, that the retreatment of calcium silicate-based sealers is more efficient than of epoxy resin sealers [37]. Still, the reason for the differences in the dislodgement resistance between the different calcium silicate-based sealer formulations is questionable. It is evident that the different compositions are related to differences in the interaction to root dentin. In the present study, a monophasic calcium silicate-based sealer showed favorable results.

With regard to the mode of failure, the present results obtained for Total Fill BC Sealer are in accordance with previously reported findings [32, 38]. Further comparisons with other investigations are not possible as either the mode of failure was not reported in studies on dislodgement resistance or the experimental setup differed from the methodology used in the present study. AH Plus and Total Fill BC Sealer predominantly showed cohesive failure modes. When considering that BioRoot RCS and Endo CPM Sealer mainly displayed mixed failure modes it is evident that higher POBS are correlated with predominantly cohesive failure modes whereas lower POBS are associated with mixed failure modes. Furthermore, it is likely that the link between epoxy resin sealers and gutta-percha is weaker than the link to dentin which can be concluded from the higher proportion of cohesive failures in the AH Plus group. The same conclusion can be made for Total Fill BC Sealer. Regarding the greater proportion of mixed failures with BioRoot RCS and Endo CPM Sealer, it can be presumed that the link between these sealers to dentin is comparable to the link to gutta-percha. The lesser proportion of adhesive failures in all groups gives evidence for a mechanism of adhesion of calcium silicate-based sealers to root canal dentin (Fig. 2).

To the best of our knowledge, the present study provides first information concerning the dislodgement resistance of BioRoot RCS. It also extends the knowledge about the dislodgement resistance of calcium silicate-based sealer under clinically relevant conditions. Regarding the different POBS values of the calcium silicate-based sealers, further evaluation of these sealers under experimental and clinical conditions is required.

## Conclusion

Within the limitations of this study (extracted teeth, age of patient, in-vitro study), the push-out bond strength of the investigated calcium silicate-based sealers was lower than the push-out bond strength of AH Plus. Total Fill BC showed the highest push-out bond strength of the calcium silicate-based sealers. BioRoot RCS displayed higher push-out bond strength than Endo CPM Sealer.

## Abbreviation

POBS: Push-out bond strength
